# A Universal Polyiodide Regulation Using Quaternization Engineering toward High Value‐Added and Ultra‐Stable Zinc‐Iodine Batteries

**DOI:** 10.1002/advs.202105598

**Published:** 2022-03-06

**Authors:** Leiqian Zhang, Mingjie Zhang, Hele Guo, Zhihong Tian, Lingfeng Ge, Guanjie He, Jiajia Huang, Jingtao Wang, Tianxi Liu, Ivan P. Parkin, Feili Lai

**Affiliations:** ^1^ School of Chemical Engineering Zhengzhou University Zhengzhou 450001 P. R. China; ^2^ Department of Chemistry KU Leuven Celestijnenlaan 200F Leuven 3001 Belgium; ^3^ Engineering Research Center for Nanomaterials Henan University Kaifeng 475004 P. R. China; ^4^ School of Chemistry University of Bristol Cantock's Close Bristol BS8 1TS UK; ^5^ Christopher Ingold Laboratory Department of Chemistry University College London 20 Gordon Street London WC1H 0AJ UK; ^6^ Key Laboratory of Synthetic and Biological Colloids Ministry of Education School of Chemical and Material Engineering International Joint Research Laboratory for Nano Energy Composites Jiangnan University Wuxi 214122 P. R. China

**Keywords:** electrostatic interaction, mechanism, quaternization, solution‐based iodine chemistry, zinc‐iodine battery

## Abstract

The development of aqueous rechargeable zinc‐iodine (Zn‐I_2_) batteries is still plagued by the polyiodide shuttle issue, which frequently causes batteries to have inadequate cycle lifetimes. In this study, quaternization engineering based on the concept of “electric double layer” is developed on a commercial acrylic fiber skeleton ($1.55–1.7 kg^−1^) to precisely constrain the polyiodide and enhance the cycling durability of Zn‐I_2_ batteries. Consequently, a high‐rate (1 C–146.1 mAh g^−1^, 10 C–133.8 mAh g^−1^) as well as, ultra‐stable (2000 cycles at 20 C with 97.24% capacity retention) polymer‐based Zn‐I_2_ battery is reported. These traits are derived from the strong electrostatic interaction generated by quaternization engineering, which significantly eliminates the polyiodide shuttle issue and simultaneously realizes peculiar solution‐based iodine chemistry (I^−^/I_3_^−^) in Zn‐I_2_ batteries. The quaternization strategy also presents high practicability, reliability, and extensibility in various complicated environments. In particular, cutting‐edge Zn‐I_2_ batteries based on the concept of derivative material (commercially available quaternized resin) demonstrate ≈100% capacity retention over 17 000 cycles at 20 C. This work provides a general and fresh insight into the design and development of large‐scale, low‐cost, and high‐performance zinc‐iodine batteries, as well as, other novel iodine storage systems.

## Graphical abstract

Herein, a universal quaternization engineering by the “electric double layer” concept is developed on commercially available polymer skeleton to precisely constrain the polyiodide. The as‐developed zinc‐iodine (Zn‐I_2_) batteries significantly eliminate the polyiodide shuttle issue and simultaneously realize solution‐based iodine chemistry (I^−^/I_3_^−^). Consequently, cutting‐edge Zn‐I_2_ batteries show ≈100% capacity retention after 17 000 cycles.

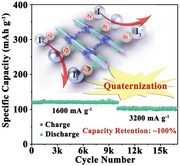

## 1 Introduction

Given the looming concerns of global warming, the development of low‐carbon energy sources such as wind and solar energy has received extensive attention worldwide, which has subsequently triggered the search for inherently safe and low‐cost electrochemical energy storage systems.^[^ ^1^, ^2^, ^3^ ^]^ Among various options, aqueous rechargeable batteries have been considered as one of the most attractive candidates because of their cost‐efficiency, eco‐friendliness, and non‐flammable features.^[^ ^4^, ^5^, ^6^ ^]^ In particular, aqueous rechargeable zinc‐iodine (Zn‐I_2_) batteries are increasingly considered because of the highly abundant components of zinc (0.0075% Zn in Earth's crust) and iodine (55 µg of iodine in every liter of seawater).^[^ ^7^, ^8^ ^]^ As an emerging metal anode, Zn can deliver a dip redox potential (−0.76 V vs standard hydrogen electrode [SHE]) and a high theoretical capacity of 820 mAh g^−1^.^[^ ^9^, ^10^, ^11^ ^]^ When coupled with an iodine cathode (I_2_/I^−^, 0.62 V vs SHE) that has a moderate theoretical capacity of 211 mAh g^−1^, the as‐assembled Zn‐I_2_ batteries present a promising theoretical energy density of ≈220 Wh kg^−1^ based on the total mass of the active materials of the cathode and anode.^[^ ^12^, ^13^, ^14^ ^]^ Moreover, compared with non‐aqueous electrolytes (≈1–10 mS cm^−1^), the aqueous electrolytes used in Zn‐I_2_ batteries normally exhibit higher ionic conductivities up to 1 S cm^−1^, which favors fast charging/discharging behaviors for real‐life needs.^[^ ^5^ ^]^ Therefore, because of the peculiarities of Zn‐I_2_ batteries with abundant resources, high energy density, high safety, and stable cycling/rate performance, they are promising alternatives to fill the gap between traditional lead‐acid batteries (≈25–50 Wh kg^−1^) and lithium‐ion batteries (≈75–200 Wh kg^−1^).^[^ ^15^, ^16^, ^17^, ^18^, ^19^ ^]^

Nonetheless, like lead‐acid and lithium‐ion batteries, Zn‐I_2_ batteries are also required to overcome their limitations before they reach the market. A reversible two‐electron redox reaction (I^−^ ↔ I_3_^−^ ↔ I_2_) occurs at the cathodes of Zn‐I_2_ cells, which is similar to a “double‐edged sword”. The high solubility of polyiodide in an aqueous electrolyte guarantees high iodine utilization as well as rapid reaction kinetics at the iodine cathode.^[^ ^7^, ^20^ ^]^ However, it also implies that polyiodide will rapidly dissolve and shuttle from the cathode, which will not only cause the loss of active materials with a rapid capacity fade but also lead to relatively low coulombic efficiency (CE). Carbon matrices (e.g., doped graphene foam,^[^ ^15^ ^]^ microporous carbon,^[^ ^21^ ^]^ and carbon fiber cloth^[^ ^22^ ^]^) have been commonly employed as I_2_ hosts to alleviate the tiresome shuttle effect of polyiodide. Unfortunately, the weak physical barriers and low chemical interactions of the carbon matrices can only slightly slow the diffusion of polyiodide. Concurrently, the chaotic pore structures of carbon matrices also lead to inadequate iodine utilization and sluggish reaction kinetics to some extent.^[^ ^23^ ^]^

Considering that the shuttle effect originates from the polyiodide anions, it may be an ideal approach to restrict the free migration of polyiodide anions and anchor them to the cathode by constructing an “electric double layer” (EDL) structure. In theory, the strong electrostatic interaction produced by the EDL structure not only provides sufficient chemical interaction to restrict polyiodide, but is also beneficial for boosting the cathodic redox reaction.^[^ ^24^, ^25^ ^]^ An ideal EDL in Zn‐I_2_ batteries is expected to contain two particular structures, that is, a cation outer layer and a water‐insoluble cation carrier inner layer. The former provides an electrostatic interaction source to couple with polyiodide anions and simultaneously suppress their migration, while the latter serves as a “port” to preserve the cation layer and avoid it becoming missing in the aqueous electrolyte. However, the establishment of an advanced EDL at the cathode of a Zn‐I_2_ battery is still lacking. Based on the essence of the EDL, organic polymer materials may be promising candidates for the construction of EDL structures in cathodes owing to their structural diversity and flexible designability.^[^ ^26^, ^27^ ^]^ By deliberately selecting the functional group and polymer skeleton, the functions of the cation layer and water‐insoluble cation carrier can be integrated into a specific organic polymer.

**Figure 1:**
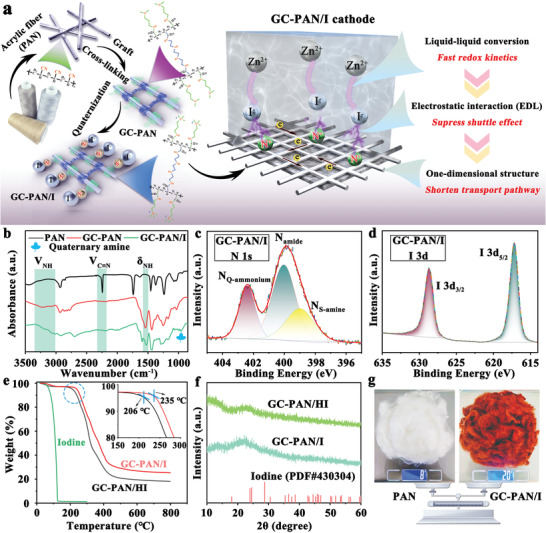
a) Schematic illustration of the synthetic process of *N,N'*‐dimethyl‐1,3‐propanediamine‐grafted and triethylenetetramine‐crosslinked acrylic fiber/iodine (GC‐PAN/I), as well as, its advantages as a cathode in zinc‐iodine (Zn‐I_2_) batteries. b) Fourier‐transform infrared (FTIR) spectra for PAN, GC‐PAN, and GC‐PAN/I. c) High‐resolution X‐ray photoelectron spectroscopy (XPS) spectra of N 1s, and d) I 3d for GC‐PAN/I. e) Thermal gravimetric analysis (TGA) curves of GC‐PAN/I, GC‐PAN/HI, and iodine. f) X‐ray diffraction (XRD) patterns of iodine, GC‐PAN/I, and GC‐PAN/HI. g) The photographs of initial PAN (8 g) and synthetic GC‐PAN/I (20 g).

In this work, a unique *N,N'*‐dimethyl‐1,3‐propanediamine‐grafted, and triethylenetetramine‐crosslinked acrylic fiber/iodine (GC‐PAN/I) cathode was prepared using in situ quaternization engineering, which realizes rational polyiodide regulation in Zn‐I_2_ batteries under the guidance of the EDL concept. A highly facile and processable two‐step reaction is conducted to develop the GC‐PAN/I cathode and form a uniform electrostatic layer that involves iodine anions and quaternary ammonium cations on the commercially available acrylic fiber skeleton (only $1.55–1.7 kg^−1^). When implemented as a cathode, the multilevel structure of GC‐PAN/I shows several peculiar properties, as illustrated in **Figure** **1**a: 1) Distinctive solution‐based cathode chemistry (I^−^/I_3_^−^) created by quaternization engineering allows Zn‐I_2_ batteries to hold high‐level redox kinetics; 2) the strong electrostatic interaction produced in the electrostatic layer significantly eliminates the polyiodide shuttle issue, simultaneously reduces the energy of the iodine reaction pathway, and results in rapid cathode chemistry in Zn‐I_2_ cells; 3) the well‐aligned 1D structure derived from the low‐cost acrylic fiber skeleton shortens the zinc ion transport pathway and promotes iodine utilization. Owing to these advantages, the Zn‐I_2_ batteries assembled by the GC‐PAN/I cathode realize superior cyclability with an ignorable capacity fading rate of 0.00138% per cycle after long‐term 2000 cycles at 20 C (1 C = 160 mAh g^−1^) and high rates (1 C–146.1 mAh g^−1^, 10 C–133.8 mAh g^−1^). Encouragingly, we also proved the high practicability and extensibility of quaternization engineering in the construction of other advanced Zn‐I_2_ batteries cathodes.

The GC‐PAN/I was synthesized through a facile two‐step reaction (Figure 1a) with acrylic fibers (PAN) as the skeleton and quaternary ammonium groups as the cation layer (see Experimental Section and Figure S1, Supporting Information, for details). Owing to the intense solvation tendency of the quaternary ammonium groups in polar solvents, a crosslinking step for PAN is essential to avoid the collapse of its skeleton. As observed in Figures S2 and S3, Supporting Information, the 1D structures of PAN were well maintained in the GC‐PAN and GC‐PAN/I, while the PAN without cross‐linking treatment was pulverized and dissolved in solution after quaternization. Furthermore, the cross‐linking reaction can slightly increase the diameter of PAN fibers from ≈17 to ≈20 µm of GC‐PAN (Figure S4a,b, Supporting Information). Allowing for the in situ quaternization between GC‐PAN and methyl iodide, the diameter of GC‐PAN/I increased to ≈25 µm (Figure S4c, Supporting Information). To be noted, methyl iodide here not only works as the methylating reagent to introduce quaternary ammonium cations on GC‐PAN, but also serves as a source of active substances (iodine). Therefore, the methyl iodide can realize 100% atom utilization to avoid the subsequent separation and purification processes, leading to the simultaneous formation of an EDL structure on GC‐PAN. The corresponding elemental mapping images of GC‐PAN/I shown in Figure S5, Supporting Information, the similar light region of each element (C, N, O, and I) indicates that an electrostatic layer composed of iodine anions and quaternary ammonium cations is uniformly formed on the PAN skeleton through quaternization engineering. Fourier‐transform infrared (FTIR) and X‐ray photoelectron spectroscopy (XPS) analyses were conducted to elucidate the synthetic mechanism of GC‐PAN/I. As shown in Figure 1b, the cyano group of PAN at 2240 cm^−1^ disappeared completely after the cross‐linking and grafting reactions. Additionally, an additional two peaks appearing at ≈3100–3300 and 1530 cm^−1^ are indicative of the stretching vibration of N—H for the secondary amine and the deformation vibration of the amide N—H, which is regarded as conditional for the success of the cross‐linking and grafting reaction. Two newly emerging peaks at 990 and 947 cm^−1^ are the characteristic peaks of the quaternary ammonium groups from quaternization between the GC‐PAN and methyl iodide.^[^ ^28^ ^]^ As shown by the XPS spectrum of N 1s for the GC‐PAN/I in Figure 1c, it can be divided into three peaks at 399.0, 400.0, and 402.4 eV, which correspond to secondary amine‐type nitrogen (N_S‐amine_), amide‐type nitrogen (N_amine_), and quaternary ammonium‐type nitrogen (N_Q‐ammonium_), respectively.^[^ ^29^ ^]^ However, a new tertiary amine‐type nitrogen appears at 401.7 eV for the GC‐PAN (Figure S6, Supporting Information), and is further replaced by the quaternary ammonium‐type nitrogen in GC‐PAN/I, which demonstrates the successful conversion of tertiary amine into quaternary ammonium through quaternization.^[^ ^30^ ^]^ Furthermore, the XPS C 1s spectra of GC‐PAN and GC‐PAN/I provided in Figure S7, Supporting Information, also support this inference. The high‐resolution I 3d spectrum of GC‐PAN/I is also presented in Figure 1d, which shows two peaks at 617.2 and 628.7 eV that correspond to I 3d_5/2_ and I 3d_3/2_, respectively.^[^ ^31^ ^]^

To further reveal the possible advantages of the EDL structure in achieving high‐performance Zn‐I_2_ batteries, GC‐PAN/hydrogen iodide (GC‐PAN/HI) was also prepared as a control sample by soaking GC‐PAN in 1 m hydrogen iodide solution to load active iodine through weak Lewis acid‐base interactions between the secondary amine and iodine. The TGA curves presented in Figure 1e show that the solid iodine disappears quickly and completely in the temperature range of 0—127 °C, while the decomposition temperatures of iodine components in both GC‐PAN/I and GC‐PAN/HI are greatly delayed. In particular, the weight loss temperature of GC‐PAN/I (235 °C), corresponding to the decomposition of amine‐type groups and the PAN chain, is relatively higher than that of GC‐PAN/HI (206 °C), which may be attributed to the stronger interactions between the iodine and quaternary ammonium groups in GC‐PAN/I. The powder XRD patterns of GC‐PAN/I and GC‐PAN/HI in Figure 1f exhibit obvious amorphous characteristics, demonstrating the existence of iodine in the form of ions rather than molecules. Given these results, we can consider the successful generation of an ideal EDL through quaternization engineering on GC‐PAN/I, which consists of iodine anions, quaternary ammonium cations, and a PAN skeleton. The iodine contents in GC‐PAN/I and GC‐PAN/HI were determined to be ≈32% and ≈40% using the argentometric and gravimetric methods, respectively. Owing to the simple two‐step reaction and the use of industrial‐scale raw materials, we synthesized over 20 g of GC‐PAN/I from 8 g of commercial PAN in a single procedure (Figure 1g). Besides, given the prices of the other main raw materials (iodine–$40.8 kg^−1^, *N,N'*‐dimethyl‐1,3‐propanediamine–$19.5 kg^−1^, triethylenetetramine–$3.06 kg^−1^), the cost of GC‐PAN/I does not exceed $25 kg^−1^. This demonstrates the tremendous potential of GC‐PAN/I as a commercially viable cathode material for zinc‐iodine batteries.

Subsequently, the constraining ability of GC‐PAN/I and GC‐PAN/HI for polyiodide species were evaluated by adding them to a series of polyiodide solutions composed of 1 m KI and 0.01 m I_2_ for an hour. Despite the preloaded iodine in both GC‐PAN/I and GC‐PAN/HI, their constraining abilities toward polyiodide were still outstanding. As shown in the digital images of polyiodide solutions and their ultraviolet‐visible (UV–vis) spectra shown in Figure S8, Supporting Information, GC‐PAN/I captured I_3_^−^ more quickly than GC‐PAN/HI, and the mass ratio (I_3_^−^ to fiber) for complete adsorption of I_3_^−^ was 1:1 for GC‐PAN/I instead of 1:2 for GC‐PAN/HI. Notably, when the dosage of GC‐PAN/I was decreased dramatically to a mass ratio (I_3_^−^ to fiber) of 1:0.25, I_3_^−^ could also be captured completely within 24 h (Figure S9, Supporting Information) because of the superiority of EDL constructed by quaternization in GC‐PAN/I to fix iodine.

**Figure 2:**
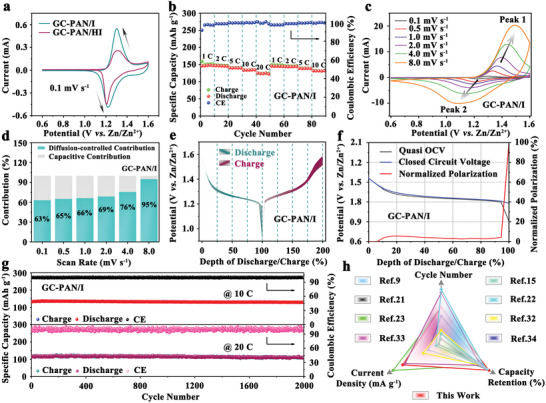
a) Cyclic voltammetry (CV) curves for *N,N'*‐dimethyl‐1,3‐propanediamine‐grafted and triethylenetetramine‐crosslinked acrylic fiber/iodine (GC‐PAN/I) and GC‐PAN/HI at a scan rate of 0.1 mV s^−1^. b) Rate performance of zinc‐iodine (Zn‐I_2_) cells based on GC‐PAN/I cathode at various current densities from 1 to 20 C. c) CV curves of GC‐PAN/I cathode at different scan rates from 0.1 to 8.0 mV s^−1^. d) Normalized ratio of capacitive contributions for a Zn‐I_2_ battery with GC‐PAN/I cathode at different scan rates. e) Galvanostatic intermittent titration (GITT) plots of Zn‐I_2_ cells using the GC‐PAN/I cathode during charge and discharge processes. f) Normalized polarization of Zn‐I_2_ cells based on the GC‐PAN/I cathode. g) Long‐term cycling performance of GC‐PAN/I cathode at 10 and 20 C. h) Comparison of cycle number, current density, and capacity retention of this work to other iodine cathodes reported in the literature.^[^ ^9^, ^15^, ^21^, ^22^, ^23^, ^32^, ^33^, ^34^ ^]^

The as‐developed GC‐PAN/I cathode was implemented in Zn‐I_2_ coin cells to evaluate its practical effect on battery performance. The cyclic voltammetry (CV) curves of the Zn‐I_2_ batteries shown in **Figure** **2**a illustrates that both the GC‐PAN/I and GC‐PAN/HI cathodes exhibit one pair of redox peaks. When compared with that of GC‐PAN/HI, the cyclic voltammetry (CV) curve of GC‐PAN/I (scan rate: 0.1 mV s^−1^) shows not only increased current but also a closer position between oxidation and reduction peaks, demonstrating its excellent redox kinetics. This observation was also confirmed by the electrochemical impedance spectroscopy (EIS) measurements in Figure S10, Supporting Information, where the cell with the GC‐PAN/I cathode shows a much smaller charge‐transfer resistance (*R*_ct_) of 71 Ω compared with that of the cell with the GC‐PAN/HI (363 Ω) cathode. The rate performance of the Zn‐I_2_ battery was evaluated under various current densities. As shown in Figure 2b, the initial discharge capacity of the GC‐PAN/I cathode is 146.1 mAh g^−1^ at 1 C (1 C = 160 mAh g^−1^), which could be well preserved at 100%, 100%, 96%, 92%, and 84% at high C‐rates of 1, 2, 5, 10, and 20 C, respectively. After reversing the current density back to 1, 2, 5, and 10 C successively, reversible discharge capacities of 145.6, 144.1, 139.0, and 131.6 mAh g^−1^ recover with about 100% capacity restorations. However, the GC‐PAN/HI cathode exhibited a more rapid capacity decline (82% from 1 to 20 C) and less capacity restoration (90% from 20 to 1 C) (Figure S11, Supporting Information) compared to GC‐PAN/I, indicating that the strong electrostatic interaction in GC‐PAN/I can result in better electrochemical performance and realize faster charging/discharging behavior than the weak Lewis acid‐base interaction in GC‐PAN/HI. Besides, the CE values of zinc‐iodine cells are relatively inferior on account of the dissolution of polyiodide into electrolytes during the longer period in each cycle at low current densities.^[^ ^7^, ^15^ ^]^ However, we still observe a high CE of GC‐PAN/I (≈97.5%) at 1 C, demonstrating its structural superiority toward zinc‐iodine batteries. Simultaneously, one significant phenomenon to note is that the GC‐PAN/I electrode shows a lower discharge capacity (146.1 mAh g^−1^) compared with that of the GC‐PAN/HI cathode (160.9 mAh g^−1^). Considering that the capacity of bare GC‐PAN without iodine is almost zero (Figure S12, Supporting Information), the capacities for GC‐PAN/I and GC‐PAN/HI can only originate from the iodine species loaded on the PAN skeleton. The energy storage mechanisms in the GC‐PAN/I and GC‐PAN/HI cathodes are completely different because of the difference between the theoretical capacities of I_2_ (211 mAh g^−1^) and I_3_^−^ (140.6 mAh g^−1^). Therefore, in terms of the discharge capacity, GC‐PAN/I cathode chemistry can be regarded as solution‐based iodine chemistry (I^−^/I_3_^−^), while it refers to a liquid‐solid process from I^−^ to I_2_ in the GC‐PAN/HI cathode chemistry. However, solution‐based reaction commonly has more rapid kinetics compared with the solid‐liquid reaction due to its larger reactive contact area, which partly explains the outstanding rate property of the GC‐PAN/I cathode.

To further understanding, the kinetic analyses were conducted through CV curves under different scan rates from 0.1 to 8.0 mV s^−1^. As shown in Figure 2c, the oxidation and reduction peaks at various scan rates are denoted as Peaks 1 and 2, indicating the formation and transformation of I^−^/I_3_^−^ during the charge and discharge processes. Subsequently, the diffusion rates of Zn^2+^ in the GC‐PAN/I cathode were evaluated using the Randles‐Sevcik equation (Equation (1)) with the data obtained from the CV curves at different scan rates.^[^ ^35^, ^36^ ^]^

(1) $$I_{p}=2.69\times 10^{5}n^{3/2}AD^{1/2}v^{1/2}C_{Zn^{2+}}$$

where *I*_p_ is the peak current (A), *n* is the number of electrons transferred in the reaction, *A* is the electrode area, *D* is the diffusion coefficient of Zn^2+^, *v* is the scan rate, and *C*_Zn2+_ is the concentration of Zn^2+^. The values of *I*_p_ and *v* ^1/2^ of GC‐PAN/I show good linear relations with slopes of 8.02 (Peak 1) and −3.82 (Peak 2), as shown in Figure S13a, Supporting Information. Therefore, the diffusion coefficients of D_Zn2+_ in GC‐PAN/I cathode are 5.44 × 10^−9^ cm^2^ s^−1^ (Peak 1) and 1.23 × 10^−9^ cm^2^ s^−1^ (Peak 2), which are about 2–5 orders of magnitude higher than the diffusion coefficients of D_Li+_ in LiFePO_4_ and LiCoO_2_ (≈10^−14^–10^−11^ cm^−2^ s^−1^).^[^ ^37^, ^38^ ^]^ According to the relationship between the measured current (*i*) and scan rate (*v*) obtained from the CV curves, the degree of the capacitive effect in the GC‐PAN/I cathode was also analyzed using Equation (2).

(2) $$i=av^{b}$$

where *a* and *b* are related to adjustable values.^[^ ^39^ ^]^ The *b*‐values of the GC‐PAN/I cathode corresponding to Peaks 1 and 2 are determined to be 0.83 and 0.68 (Figure S13b, Supporting Information), respectively, suggesting a combined contribution of diffusion and capacitive processes for the cathode chemistry. The capacitive (*k*_1_ *v*) and diffusion contributions (*k*_2_ *v* ^1/2^) in the GC‐PAN/I cathode were further quantified according to Equations (3) and (4).^[^ ^40^, ^41^ ^]^

(3) $$i=k_{1}v+k_{2}v^{1/2}$$

(4) $$i/v^{1/2}=k_{1}v^{1/2}+k_{2}$$

As a result, the capacitive part accounts for 63% at a scan rate of 0.1 mV s^−1^, which increased to 95% at a scan rate of 8 mV s^−1^ (Figure 2d). The capacitance‐dominant process in the GC‐PAN/I cathode is one of the main reasons for the ultra‐fast charging behavior of the assembled Zn‐I_2_ batteries.^[^ ^35^ ^]^ Additionally, galvanostatic intermittent titration experiments were conducted to investigate the kinetics of the GC‐PAN/I cathode at a low current of 60 µA. As plotted in Figure 2e, the shadow parts between the charging‐discharging curves corresponding to the degree of polarization in the GC‐PAN/I cathode present a minor area. Furthermore, the degree of polarization was quantified by introducing the internal resistance (*R*_internal_) based on the following equation:^[^ ^42^ ^]^

(5) $$\Delta R_{\mathrm{internal}}\left(\Omega \right)=\left|\Delta V_{\mathrm{QOCV}-\mathrm{CCV}}\right|/I_{\mathrm{applied}}$$

where Δ*V*_QOCV‐CCV_ represents the voltage difference between the points of quasi open circuit voltage (QOCV) and closed circuit voltage (CCV), and *I*_applied_ refers to the applied current. At this point, GC‐PAN/I shows a low resistance until about 94.5% of the depth of discharge after normalized treatment for the polarization curve (Figure 2f), which demonstrates that the Zn^2+^ diffusion behavior is fairly rapid in Zn‐I_2_ cells with GC‐PAN/I cathode.^[^ ^43^ ^]^ Overall, the superior rate capability of the GC‐PAN/I cathode is ascribed to the fast Zn^2+^ diffusion behavior of solution‐based iodine chemistry (I^−^/I_3_^−^) and the capacitance‐dominant process during the charge and discharge processes.

Long‐term cycling tests were performed to evaluate the stability of the two cathodes. In stark contrast to the rapid battery failure observed for the GC‐PAN/HI cathode (only 67.95% capacity retention after 2000 cycles, Figure S14, Supporting Information), the Zn‐I_2_ battery using the GC‐PAN/I cathode displays superior cycling performance with a high capacity retention of 98.71% and a reversible capacity of 130.5 mAh g^−1^ after 2000 cycles at 10 C (Figure 2g). The corresponding galvanostatic discharge/charge profiles of GC‐PAN/I at 10 C are also provided in Figure S15, Supporting Information. When the current density is increased to 20 C, the Zn‐I_2_ cells with the GC‐PAN/I electrode are ultra‐stable with a capacity retention of 97.24%, corresponding to a reversible capacity of 112.6 mAh g^−1^ after 2000 cycles. The near‐perfect stability and excellent rates of the GC‐PAN/I cathode fully prove the success of quaternization engineering based on the EDL concept. To gain a better understanding of this study, some representative works were further compared on different dimensions from cycle number, current density, and capacity retention, as shown in Figure 2h (the related literature values can be obtained in Table S2, Supporting Information). As expected, the performance of the GC‐PAN/I cathode is exceptional in every aspect, especially in terms of stability. Using industrial‐scale raw materials with a facile two‐step synthesis path, the GC‐PAN/I cathode could become a competitive cathode material for the realization of advanced Zn‐I_2_ battery systems.

**Figure 3:**
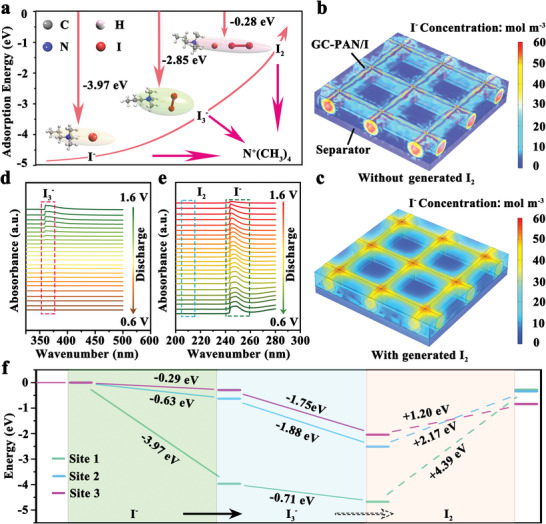
Reaction mechanism for zinc‐iodine (Zn‐I_2_) battery by using *N,N'*‐dimethyl‐1,3‐propanediamine‐grafted and triethylenetetramine‐crosslinked acrylic fiber/iodine (GC‐PAN/I) as the cathode. a) The optimized structures and the values of binding energies for I^−^, I_3_^−^, and I_2_ in Site 1. The concentration distribution diagrams of I^–^ in a Zn‐I_2_ cell following different reaction paths of b) from I_3_^−^ to I^−^, and c) from I_2_ to I^−^ during the discharge process. d,e) In situ ultraviolet‐visible (UV–vis) analysis of GC‐PAN/I electrode during the discharge process. f) The calculated energies for the process of iodine species oxidation reactions on active centers of Sites 1, 2, and 3.

Furthermore, root cause analysis based on density functional theory (DFT) calculations were conducted to understand the underlying mechanism of quaternization engineering in realizing high‐performance Zn‐I_2_ batteries. To simplify the calculation reasonably, GC‐PAN/I was divided into quaternary ammonium centers (Site 1), amide centers (Site 2), and secondary amine centers (Site 3) (Figure S16, Supporting Information). As shown in **Figure** **3**a, the quaternary ammonium center (Site 1) as the subject of building EDL presents the binding energies of −3.97, −2.85, and −0.28 eV for I^−^, I_3_^−^, and I_2_, respectively. This indicates that the dissolved I^−^ and I_3_^−^ species are well constrained by Site 1, while the capture of I_2_ is not easy for quaternary ammonium centers. Contrastingly, Sites 2 and 3 present undesirable constraining abilities toward all of the iodine species (I^−^, I_3_^−^, and I_2_) with high binding energies (>−0.90 eV), as displayed in Table S1, Supporting Information. As Sites 2 and 3 are the two main groups in the GC‐PAN/HI cathode, these DFT calculation results also confirm their inferior electrochemical performance from a theoretical perspective. Therefore, the quaternary ammonium center is considered the dominant active site that limits the migration of iodine species (I_3_^−^ and I^−^).

Nonetheless, the relatively poor constraining ability toward I_2_ at Site 1 correspondingly could cause an unforeseeable shuttle effect during cycling, which is further expounded by COMSOL multiphysics simulations with details for geometric constructions shown in Figure S17, Supporting Information. As shown in Figure 3b, we first assumed that there was no solid I_2_ generation in the GC‐PAN/I cathode during the charging process. Therefore, the cathode chemistry of GC‐PAN/I will only refer to a conversion between I_3_^−^ and I^−^, which also means that I^−^ as the final discharge product can be well confined in the GC‐PAN/I cathode because of the strong interaction between the quaternary ammonium groups and iodine species (I_3_^−^ and I^−^). On the contrary, once solid I_2_ is generated, I_2_ will sluggishly detach from GC‐PAN/I because I_2_ is slightly soluble in water, as well as the poor constraining ability of GC‐PAN/I toward I_2_, which means that the final discharge products (I^−^) will penetrate into the electrolyte, as shown in Figure 3c. Combining the electrochemical analysis discussed previously, we deduce that the cathode chemistry of GC‐PAN/I is simply solution‐based iodine chemistry (I^−^/I_3_^−^). The in situ UV–vis spectroscopy was conducted to determine the conversion mechanism of iodine species in the GC‐PAN/I cathode. As shown in Figure 3d, the absorption peak of the GC‐PAN/I cathode in the UV–vis spectrum at ≈363 nm is associated with I_3_^−^,^[^ ^31^ ^]^ which is legible in the initial stages of the discharge process. As the discharge process continues, the intensity from the adsorption peak of I_3_^−^ reduces gradually, and even disappears as the cell potential is close to 0.6 V. Furthermore, the concentration of I^−^ gradually rises during the discharge process, as shown by the adsorption peaks at 244 nm (Figure 3e),^[^ ^44^ ^]^ and reaches its maximum level at a cell potential of 0.6 V. These results imply that I_3_^−^ is converted into I^−^ as the discharge process proceeds at the GC‐PAN/I cathode. No peaks at ≈210 nm were observed, suggesting that the discharge process in the GC‐PAN/I cathode did not generate any I_2_.^[^ ^45^ ^]^ Meanwhile, the ex situ XPS spectroscopy in Figure S18, Supporting Information, further demonstrated this point. The peaks at 620.7 and 632.2 eV can be assigned to I_3_^−^, while those at about 619.3 and 630.8 eV are associated with I^−^.^[^ ^46^ ^]^ With discharging, the peaks of I_3_^−^ become weak and finally even disappear. For the peak of I^−^, however, it gradually becomes a single one. These observations well cohere with the results of UV–vis spectroscopy, namely the solution‐based cathode chemistry from I_3_^−^ to I^−^. On this basis, we fully consider that solution‐based iodine chemistry is produced in GC‐PAN/I, which can significantly eliminate the issue of I_2_ dissolution and diffusion at the GC‐PAN/I electrode.

To understand the formation mechanism of solution‐based iodine chemistry, DFT calculations were used to evaluate the difficulty of the conversion reactions of iodide species (I^−^ → I_3_^−^ → I_2_) at three active sites. As plotted in Figure 3f, all three sites show large energy barriers for the conversion from I_3_^−^ to I_2_ (Site 1: 4.39 eV; Site 2: 2.17 eV; Site 3: 1.20 eV). This observation provides compelling theoretical evidence for the failed detection of I_2_ using in situ UV–vis spectroscopy (Figure 3e) and proves that only solution‐based iodine chemistry (I^−^/I_3_^−^) occurred in the GC‐PAN/I cathode. Simultaneously, the relatively low energy barriers of Sites 2 and 3 (the main active groups on GC‐PAN/HI) also explain why I_2_ was observed in GC‐PAN/HI (Figure S19, Supporting Information), as well as, the slightly higher capacity of GC‐PAN/HI than GC‐PAN/I. It is also noteworthy that these sites present a unified negative binding energy for the I^−^ and I_3_^−^ species. In particular, Site 1 possesses the most negative binding energy toward I^−^ (−3.97 eV) and I_3_^−^ (−4.68 eV), indicating that the strong electrostatic interaction favors the reduction of the energy of the iodine reaction pathway. This tends to result in a faster rate performance of GC‐PAN/I than GC‐PAN/HI.

**Figure 4:**
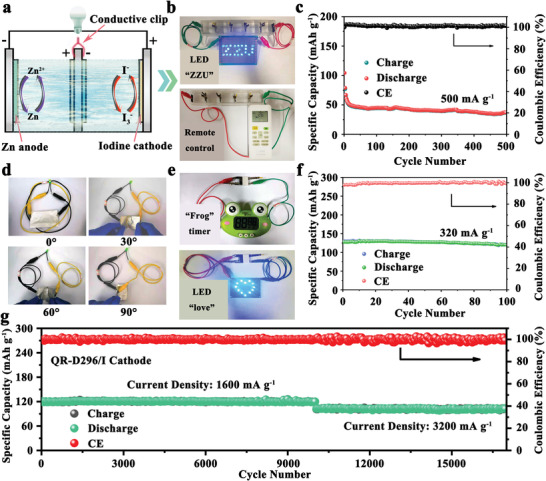
a) Schematic illustration for the open structure of zinc‐iodine (Zn‐I_2_) batteries. b) Optical image of Zn‐I_2_ batteries with open structure connected in series powering a light‐emitting diode (LED) with “ZZU” and a remote control. c) The corresponding electrochemical performance of Zn‐I_2_ batteries with an open structure. d) The images of quasi‐solid‐state *N,N'*‐dimethyl‐1,3‐propanediamine‐grafted and triethylenetetramine‐crosslinked acrylic fiber/iodine (GC‐PAN/I)‐based Zn‐I_2_ device at different bending degrees of 0°, 30°, 60°, and 90°. e) Series‐connected Zn‐I_2_ batteries based on liquid electrolyte powering timers and an LED as “love,” and f) the corresponding electrochemical performance of an individual cell. g) Long‐term cyclability of QR‐D296/I cathode at different C‐rates.

To examine the practical viability of the as‐developed GC‐PAN/I cathode, Zn‐I_2_ batteries were assembled under different conditions. As two important parameters that affect the performance of the battery, the high‐/low‐temperature tolerance and self‐discharge behavior of Zn‐I_2_ batteries with a GC‐PAN/I cathode were first studied, as shown in Figure S20a,b, Supporting Information. When the temperature increases from 25 to 60 °C, the Zn‐I_2_ cell has no significant capacity decline, while the cell exhibits a relatively large capacity loss of 35.69% (81.8 mAh g^−1^) at 0 °C in comparison with that at 60 °C (127.2 mAh g^−1^) owing to the high freezing point characteristics of the aqueous electrolyte. Despite this, Zn‐I_2_ cells using GC‐PAN/I as the cathode are still able to function properly over 100 cycles from 0 to 60 °C at 10 C. The self‐discharge behavior of Zn‐I_2_ batteries using a GC‐PAN/I cathode was also significantly suppressed. With different resting times of 5, 10, and 15 days, the battery exhibited high capacity retentions of 92.65%, 87.94%, and 85.37%, respectively. In particular, when recharging after 15 days, the cell still functions properly with a small decline in capacity of 0.78%, implying that iodine is strongly immobilized in the GC‐PAN/I cathode through quaternization engineering. To reflect the high reliability, intrinsic safety, and remarkable environmental tolerance of the GC‐PAN/I‐based Zn‐I_2_ batteries, we connected the Zn‐I_2_ batteries in series in an open environment, as illustrated in **Figure** **4**a. The Zn‐I_2_ cells are capable of lightening “ZZU” light‐emitting diodes (LED) as well as taking responsibility for the normal operation of a remote control (Figure 4b). Such a useful Zn‐I_2_ battery system can operate steadily for more than 500 cycles at a density current of 500 mA g^−1^ (Figure 4c), which optimally fits our pursuit of scale. Subsequently, a quasi‐solid‐state GC‐PAN/I‐based Zn‐I_2_ device was fabricated using a gelatin electrolyte. As shown in Figure 4d, the battery can work well at different flexures from 0° to 90°, and the corresponding cycling performance is shown in Figure S21, Supporting Information, which exhibited a stable specific capacity of ≈90 mAh g^−1^ when the bending angle changed from 0° to 90°, indicating its excellent anti‐deformation ability. In particular, series‐connected Zn‐I_2_ batteries were further assembled based on the GC‐PAN/I cathode in an easy‐to‐operate method (the detailed parameters are provided in the Supporting Information). As shown in Figure 4e, series‐connected batteries can comfortably manage timers and LED lights to work properly. Moreover, such an individual cell shows a moderate capacity of 119.6 mAh g^−1^ at 2 C after 100 cycles with an ultra‐high capacity retention of 93.73% (Figure 4f). Based on this, the energy density of the cell calculated by the active materials is ≈159.5 Wh kg^−1^, indicating that the gap between lead‐acid batteries and lithium‐ion batteries, as mentioned previously, is hopefully filled by GC‐PAN/I‐based Zn‐I_2_ batteries owing to its moderate energy density and excellent operability.

As a proof‐of‐concept, we also studied other commercially available products (the strong base resin‐D296, ≈$2.6 kg^−1^) with rich quaternary ammonium groups. Compared with the GC‐PAN/I cathode, the preparation process of the quaternized strong base resin‐D296/iodine cathode (QR‐D296/I) is simpler and can be obtained directly after soaking in polyiodide solution (see Supporting Information for details). As shown in Figure S22, Supporting Information, the CV curves of the QR‐D296/I cathode are similar to those of GC‐PAN/I. However, compared with the GC‐PAN/I cathode, the QR‐D296/I cathode exhibited a relatively poor performance, which only maintained a 71.17% capacity retention as the C‐rate increased from 1 to 20 C (Figure S23, Supporting Information). This may be ascribed to the dense structure of the QR‐D296/I cathode (Figure S24a,b, Supporting Information), which restricts the transfer of Zn^2+^. This can also be well demonstrated from the EIS measurements of the QR‐D296/I cathode (≈325 Ω) in Figure S25, Supporting Information. Remarkably, these results would seem to reflect the importance of the 1D structure for GC‐PAN/I in realizing high‐rate Zn‐I_2_ batteries. Regardless of this, the long‐term stability tests of the QR‐D296/I cathode were performed at different current densities, as shown in Figure 4g. Unexpectedly, the QR‐D296/I cathode stood out so that it showed ≈100% capacity retention with a reversible capacity of 120.7 mAh g^−1^ at 10 C after 10 000 cycles, and was able to keep cycling over 7000 cycles corresponding to ≈100% capacity retention and a reversible capacity of 105.1 mAh g^−1^ when increasing current density to 20 C. The corresponding galvanostatic discharge/charge profiles of QR‐D296/I at 10 C are also provided in Figure S26, Supporting Information. Such excellent stability of QR‐D296/I indicates the complete avoidance of polyiodide shuttling in Zn‐I_2_ batteries. It is worth noting that this is conducive to the stability of the zinc anode. As shown in Figures S27 and S28, Supporting Information, when the electrolyte contains polyiodide, zinc anodes will suffer rapid failure due to the corrosion and passivation of polyiodide toward zinc anode. All in all, this near‐perfect result fully proves the feasibility and superiority of our strategy, which could greatly promote the industrial development of Zn‐I_2_ batteries in the future.

In conclusion, a unique quaternization engineering based on the concept of EDL was developed to accurately constrain the shuttle effect of polyiodide toward low‐cost, durable, and highly operable Zn‐I_2_ batteries. The as‐developed cathode materials can provide sufficient electrostatic interaction to immobilize polyiodide to the cathode as well as reduce the reaction energy, leading to enhanced cycling durability and rate performance of Zn‐I_2_ batteries. More importantly, such quaternization engineering enables the generation of a large energy barrier between I_3_^−^ and I_2_, blocking the production of solid I_2_, thus resulting in solution‐based iodine chemistry (I^−^/I_3_^−^). Accordingly, the GC‐PAN/I‐based Zn‐I_2_ batteries present ultra‐stable performance with ≈97.24% capacity retention after 2000 cycles at 20 C and high rates (1 C–146.1 mAh g^−1^, 10 C–133.8 mAh g^−1^). Furthermore, the practical testing of GC‐PAN/I in various complicated environments demonstrates the high environmental tolerance, reliability, and moderate energy density (159.5 Wh kg^–1^) of quaternization engineering. In particular, quaternization engineering also exhibits good universality, where QR‐D296/I shows a cutting‐edge performance of ≈100% capacity retention over 17 000 cycles. This work provides a promising avenue for the design and development of large‐scale, low‐cost, and high‐performance Zn‐I_2_ batteries, which are implantable to inhibit the shuttle effect in other metal‐iodine/sulfur battery systems through the “quaternization” concept.

## Conflict of Interest

The authors declare no conflict of interest.

## Supporting information

Supporting Information

## Data Availability

The data that support the findings of this study are available from the corresponding author upon reasonable request.
