# Supplementary material for: A Universal Polyiodide Regulation Using Quaternization Engineering toward High Value‐Added and Ultra‐Stable Zinc‐Iodine Batteries
Source: Adv Sci (Weinh). 2022 Mar 6;9(13):2105598. doi: 10.1002/advs.202105598 (PMC9069388; doi:10.1002/advs.202105598)
Supplement: Supplementary file 1 — Supporting Information [file ADVS-9-2105598-s001.pdf]

Supporting Information

A Universal Polyiodide Regulation Using Quaternization Engineering toward High Value-Added and Ultra-Stable Zinc-Iodine Batteries

Leiqian Zhang, Mingjie Zhang, Hele Guo, Zhihong Tian, Lingfeng Ge, Guanjie He, Jiajia Huang, Jingtao Wang, Tianxi Liu, Ivan P. Parkin, Feili Lai**

L. Zhang, M. Zhang, Prof. J. Huang, Prof. J. Wang
School of Chemical Engineering, Zhengzhou University, Zhengzhou 450001, P. R. China
E-mail: huangjiajia@zzu.edu.cn

H. Guo, Dr. F. Lai
Department of Chemistry, KU Leuven, Celestijnenlaan 200F, Leuven 3001, Belgium
Email: feili.lai@kuleuven.be

Z. Tian
Engineering Research Center for Nanomaterials, Henan University, Kaifeng 475004, P. R. China.

L. Ge
School of Chemistry, University of Bristol, Cantock's Close, Bristol, BS8 1TS, UK

Dr. G. He, Prof. I. P. Parkin
Christopher Ingold Laboratory, Department of Chemistry, University College London, 20 Gordon Street, London WC1H 0AJ, UK

Prof. T. Liu
Key Laboratory of Synthetic and Biological Colloids, Ministry of Education, School of Chemical and Material Engineering, International Joint Research Laboratory for Nano Energy Composites, Jiangnan University, Wuxi, 214122, P. R. China

Experimental Section:

1. Materials Synthesis and Characterization:

Materials: Triethylenetetramine (99%), $ZnSO_4$ (99.5%), $AgNO_3$ (99.7%) and *N,N'*-dimethyl-1,3-propanediamine (99%) were purchased from Tianjin Kemiou Chemical Reagent Co. Ltd. *N*-methyl-2-pyrrolidinone (NMP, 99.5%), and hydroiodic acid solution (HI, 55.0-58.0%) were purchased from Shanghai Aladdin Biochemical Technology Co., Ltd. Methyl iodide (99%, AIKE Chem.), acrylic fiber (PAN, Industrial, Shandong Tech.), and resin (D296, Industrial, Langfang Miaoyang chem.) were purchased from different technology firms.

Synthesis of GC-PAN: *N,N'*-dimethyl-1,3-propanediamine-grafted, and triethylenetetramine-crosslinked acrylic fiber (GC-PAN) was synthesized through a facile one-step reaction. In short, 1.0 g of PAN fiber was firstly grafted with 35.7 g of *N,N'*-dimethyl-1,3-propanediamine, and simultaneously crosslinked with 1.7 g of triethylenetetramine at 135 °C under reflux for 12 h. Whereafter, the product was washed with deionized water and ethanol, and then vacuum-dried at 60 °C for 12 h.

Synthesis of GC-PAN/I and GC-PAN/HI: *N,N'*-dimethyl-1,3-propanediamine-grafted, and triethylenetetramine-crosslinked acrylic fiber/iodine (GC-PAN/I) was synthesized through the quaternization reaction between methyl iodide and GC-PAN. Briefly, 1.0 g of GC-PAN was firstly immersed in 22.2 g of *N,N'*-dimethylformamide (DMF), and then 1.4 g of methyl iodide was added into the solution. The reaction mixture was heated to 40 °C under reflux and kept for 8 h. Subsequently, the product was washed with deionized water and ethanol to remove the soluble substance. After that, GC-PAN/I can be obtained by vacuum drying at 60 °C for 12 h. For the GC-PAN/HI, it was synthesized by soaking GC-PAN in a 1 M HI solution (100 mL) for 12 h.

Synthesis of QR/I: The commercial quaternized strong base resin-D296 (QR-D296) was firstly washed with ethyl alcohol, 5% HCl, and 5% NaOH, respectively, to remove impurities. Then, the QR-D296, iodine, and potassium iodide were immersed in deionized water in a mass ratio of 1:0.4:33 for 12 h to load active iodine. After washing with deionized water and ethyl alcohol, respectively, the QR-D296/I can be obtained.

Materials and structural characterizations: The scanning electron microscopy (SEM) was conducted by 7500F, JEOL. The Fourier transform infrared spectroscopy (FTIR) (Bruker VERTEX 70v) spectra were recorded using KBr pellets. Thermal gravimetric analysis (TGA) (NETZSCH STA 2500 simultaneous thermal analyzer) was performed with a heating rate of 10 °C min^{-1} from room temperature to 800 °C under nitrogen atmosphere. Powder X-Ray diffraction (XRD) patterns of these fibers were recorded on a D8 ADVANCE X-ray diffractometer with Cu Ka radiation (λ =1.5406 Å). X-ray photoelectron spectroscopy (XPS) analyses were performed based

on VG ESCALAB 220I-XL. Ultraviolet-visible spectroscopy (UV-vis) (Shimadzu UV-2450 spectrometer) analyses were used to characterize iodine species in deionized water.

Electrochemical characterization: The fibers, polyvinylidene difluoride (PVDF), and Super P were mixed in a mass ratio of 80:10:10, and as-prepared slurries were further coated onto carbon paper with NMP as the solvent and an iodine loading of 1.5−2.0 mg cm^{-2}. Then, they were vacuum-dried at 45 °C for 24 h. The CR2032-type coin cells were assembled in the air by using Zn foil, glass fiber membrane, and 2 M $ZnSO_4$ as counter electrode, separator, and electrolyte, respectively. The galvanostatic charge/discharge performance (LAND-CT2001A) of the half-cell was conducted in a voltage range of 0.6−1.6 V (*vs.* Zn/Zn^{2+}) at different temperatures. Cyclic voltammetry (CV) curves were carried out on CHI660E electrochemical test station with scan rates from 0.1 to 8 mV s^{-1} in the range of 0.6−1.6 V. Electrochemical impedance spectrum (EIS) was measured on CHI660E electrochemical test station in the frequency range of 0.01 Hz to 1 MHz with alternating current (AC) amplitude of 10 mV. Galvanostatic intermittent titration (GITT) experiments were also conducted at a current of 60 μA. Specific capacities were calculated based on the mass of iodine.

Chemical titration experiments: The iodine content in the GC-PAN/I was determined by argentometry. In short, 0.2 g of fiber was firstly immersed in 100 mL of 1 M Na_2SO_4 solution for 12 h at 45 °C in the constant temperature incubator shaker. After filtering the fiber, there solutions (each of 25 mL) were obtained and titrated with excess $AgNO_3$ (0.0988 mol L^{-1}) to calculate the content of iodine by taking the average. In detail, the content of iodine (~32%) was determined by back-titration method with NH_4SCN as titrant and $NH_4Fe(SO_4)_2·12H_2O$ as the indicator. For the GC-PAN/HI and QR-D296/I, the iodine contents was calculated through the mass difference before and after adsorption (~40 wt%).

Adsorption Experiments: A polyiodide solution was synthesized by dissolving I_2 and KI in deionized water at a stoichiometric ratio of 1:100 for a total concentration of 1.5 mg mL^{-1}. Subsequently, the fiber powder was immersed in 7 mL of polyiodide solution in different mass ratios as Figure S8 for 1 h. The UV-vis spectra of the soaking solutions were performed at wavelengths from 300 to 900 nm.

In-situ UV-vis tests: For the in-situ UV-vis tests, the spectroscopy measurements were used to check the conversion of iodine in a transparent two-electrode system under a current of 100 μA at wavelengths from 200 to 500 nm. During the observation, the scanning interval of measurements was set at three minutes.

Construction of flexible battery: Firstly, the iron mesh was immersed in ethanol for ultrasonic treatment with 1 h. The GC-PAN/I was loaded on a current collector of iron mesh, and the iodine loading on the iron mesh is about 1.0 mg cm^{-2}. Then, a hydrogel electrolyte was obtained by the method reported in the literature.[1] In short, 5 g of

gelatin was added to 0.1 M $ZnSO_4$ and then stirred to clear at 60 °C. The quasi-solid-state flexible batteries were further constructed using GC-PAN/I frameworks as the cathode, Zn foil as the anode, and hydrogel as the electrolyte with a sandwich structure.

Series-connected battery assembly process: Firstly, the iron mesh was immersed in ethanol for ultrasonic treatment with 1 h. Then, the GC-PAN/I was coated to the iron mesh by doctor blade technology and dried at 45 °C for 24 h. The iodine loading on the iron mesh is about 1.0 mg cm^{-2}. Next, the iron meshes with GC-PAN/I and Zn foil were attached to two pieces of glass, respectively. Furthermore, Zn-I_2 batteries were assembled into a sandwich structure by using GC-PAN/I as the cathode, filter paper as the separator, Zn foil as the anode, and 2 M $ZnSO_4$ as the electrolyte. The batteries were connected in series with iron clamps.

Density functional theory (DFT) calculations: All quantum chemical calculations, including geometry optimizations and single-point energy calculations, were performed at the PBE1PBE[2]/def2svp[3,4] level with the D3 version of Grimme's dispersion with Becke-Johnson damping[5] in the quantum chemical package Gaussian 16[6]. The monomer of this polymer was divided into three slices, as shown in Figure S16, for the calculations of reactions at the three sites, respectively.

COMSOL Multiphysics simulations: Based on COMSOL Multiphysics 5.6 software, a 3D Zn-I_2 battery model was established according to previous reports.[7,8] In short, the "Tertiary current distribution" interface was selected to describe the current and potential distributions in the cell and analyze the transport of iodine species in the electrolyte, while Butler-Volmer expression was further used to reveal the electrode kinetics of the charge transfer reaction at room temperature in the bulk electrolyte. Considering the fast kinetics of the Zn anode, it was replaced with the surface of the separator. The detailed geometric construction is shown in Figure S17.

Part I: Supporting Figures

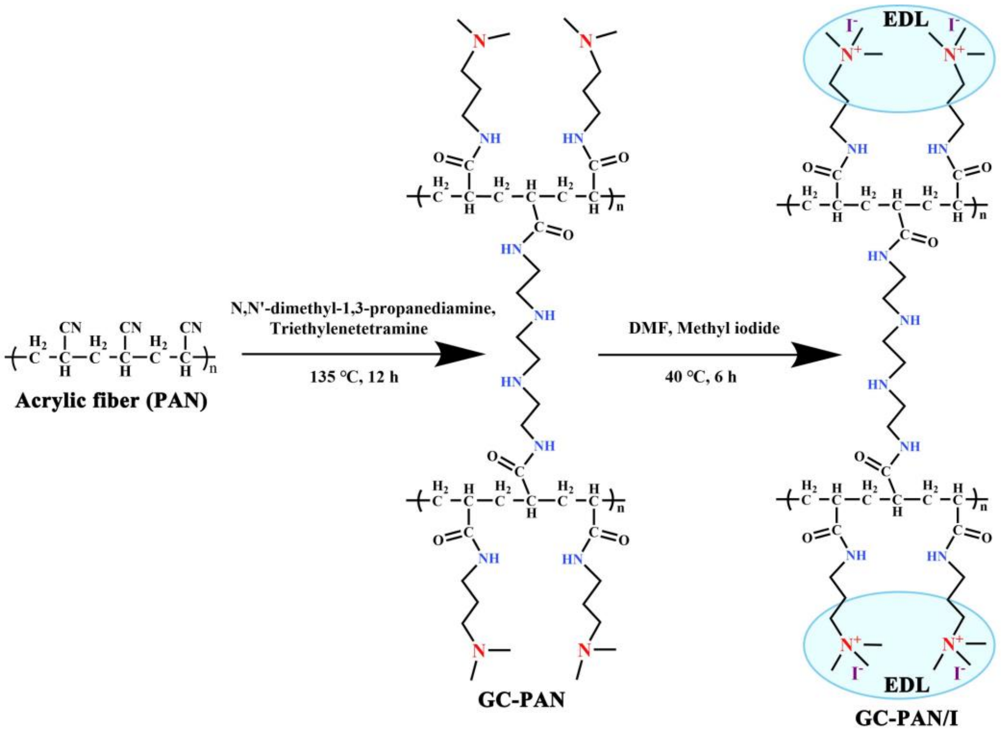

Figure S1. The schematic detailed the synthetic route for polymer-based cathode preparation. With PAN as the raw material, GC-PAN can be prepared through cross-linking and grafting reaction. Furthermore, given the methyl iodide as the alkylating reagent, the EDL structure can be formed on the GC-PAN to obtain GC-PAN/I.

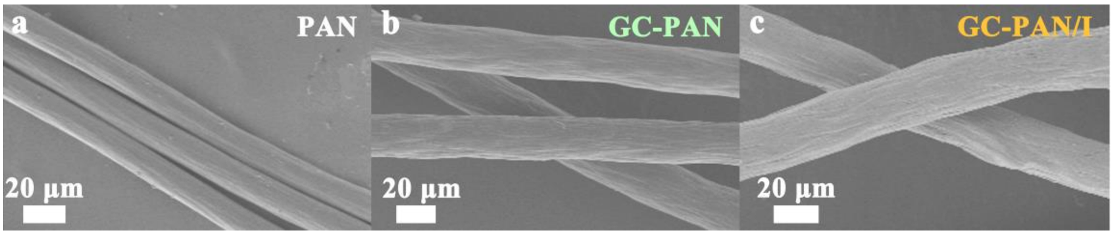

Figure S2. SEM images of a) PAN, b) GC-PAN, and c) GC-PAN/I.

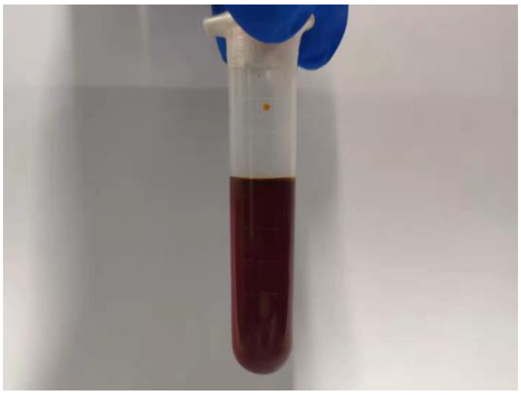

Figure S3. Digital image of PAN without cross-linking treatment after quaternization.

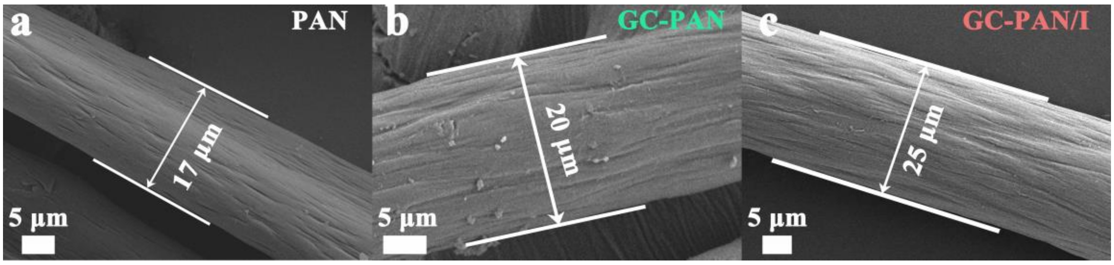

Figure S4. SEM images of a) PAN, b) GC-PAN, and c) GC-PAN/I.

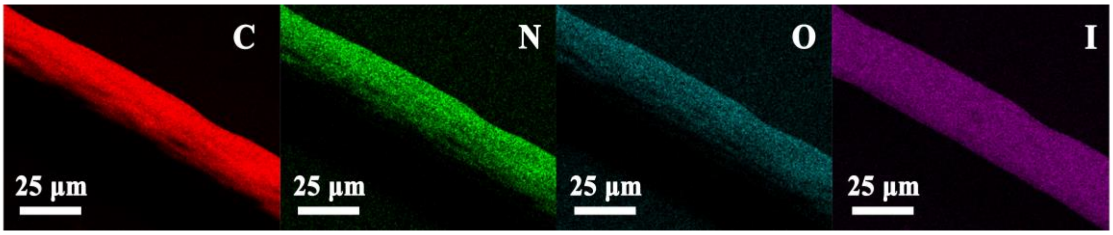

Figure S5. Elemental mapping images of C, N, O, and I elements in GC-PAN/I.

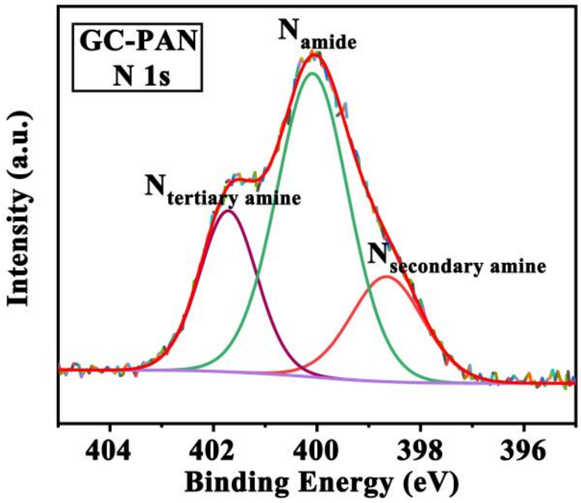

Figure S6. The high-resolution N 1s XPS spectrum of GC-PAN, indicating the successes of the grafting and crosslinking reactions.

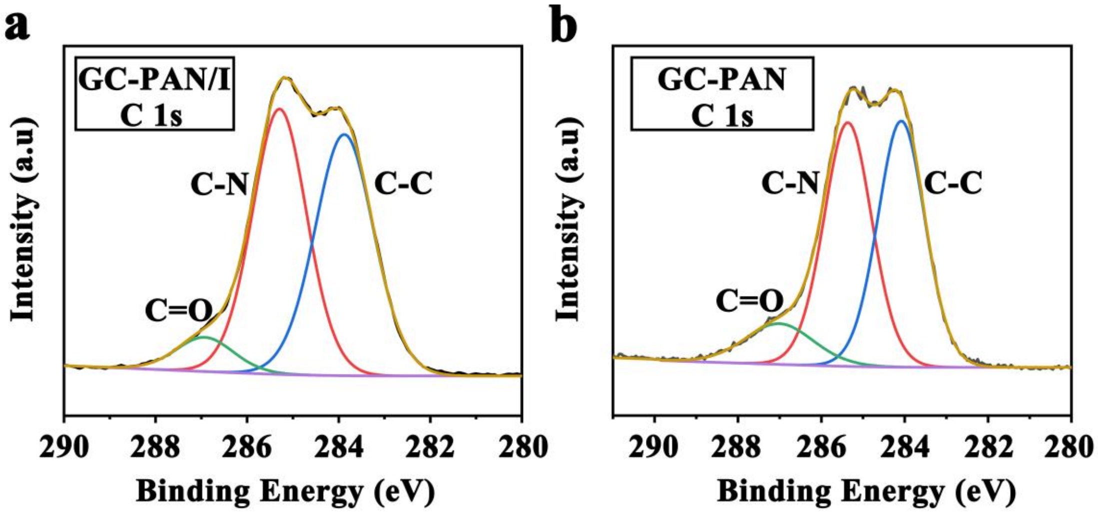

Figure S7. The high-resolution C 1s XPS spectra of a) GC-PAN/I and b) GC-PAN. The spectra were deconvoluted into C-C, C-N, and C=O.

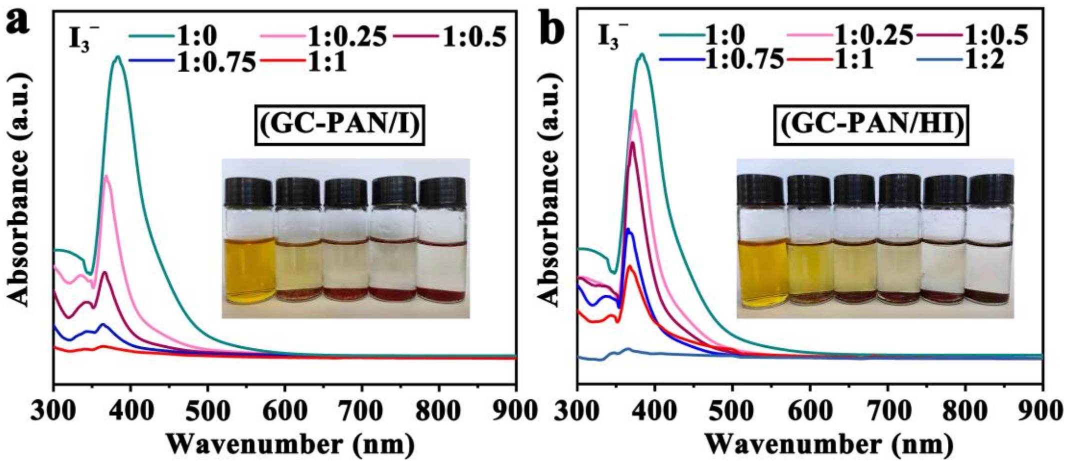

Figure S8. UV-vis spectra and digital images with different loading amounts of a) GC-PAN/I, and b) GC-PAN/HI in a series of polyiodide solutions with fixed concentrations for 1 h.

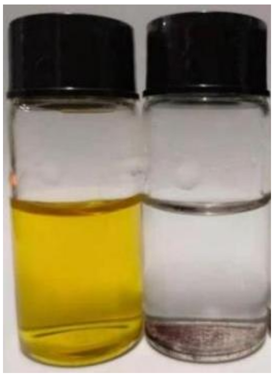

Figure S9. The photograph of polyiodide solutions without fiber (left) and with GC-PAN/I (1:0.25) (right) after standing for 24 h.

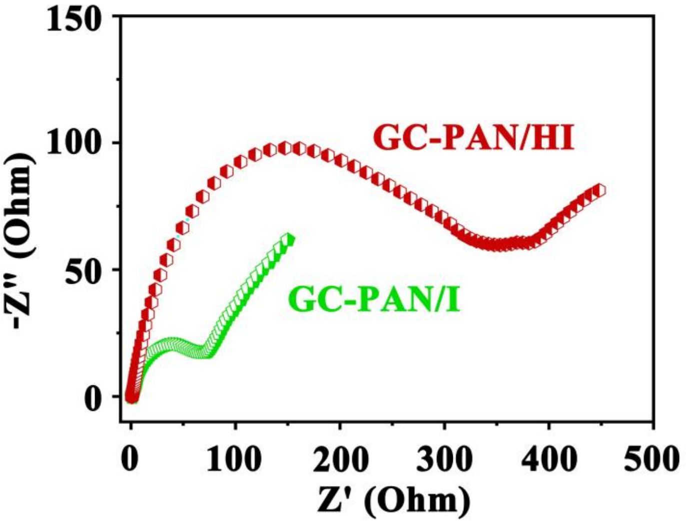

Figure S10. Electrochemical impedance spectroscopy measurements for the zinc-iodine cells with the GC-PAN/I and GC-PAN/HI cathodes.

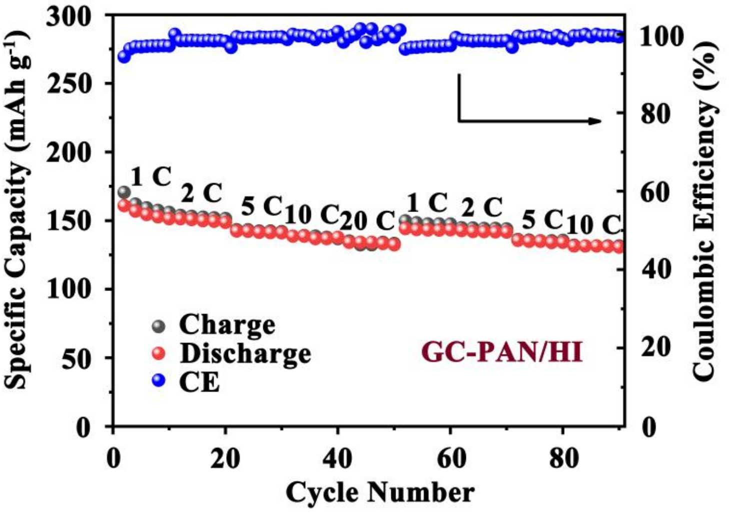

Figure S11. Rate performance of the GC-PAN/HI electrode at different C-rates.

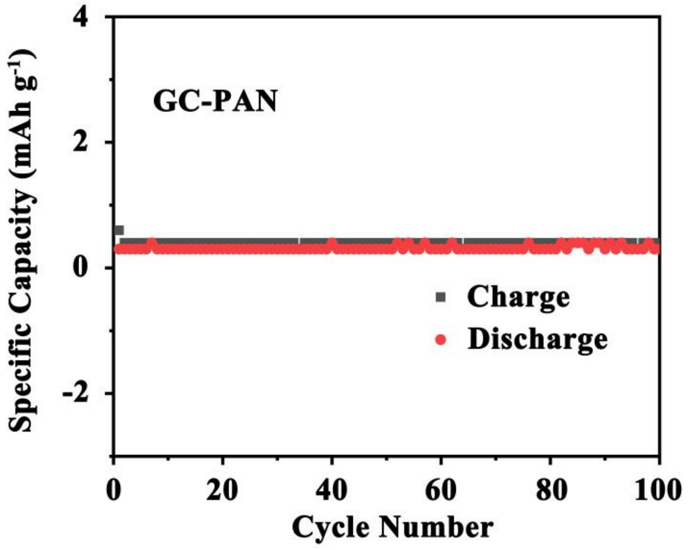

Figure S12. Cycling performance of the GC-PAN electrode at 1 C.

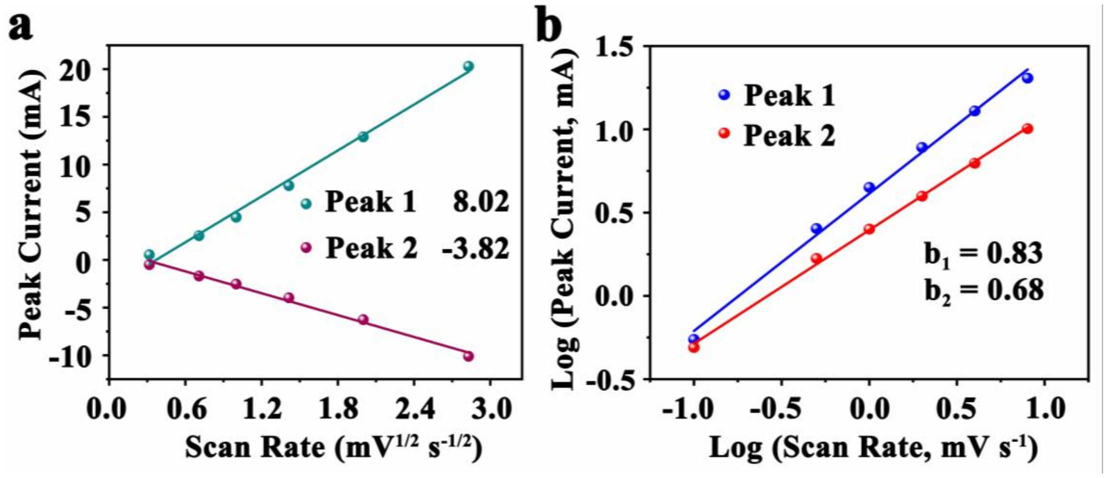

Figure S13. The relationships a) between logarithm redox peak current and logarithm scan rate, and b) between peak current and scan rate in zinc-iodine cells with GC-PAN/I electrode.

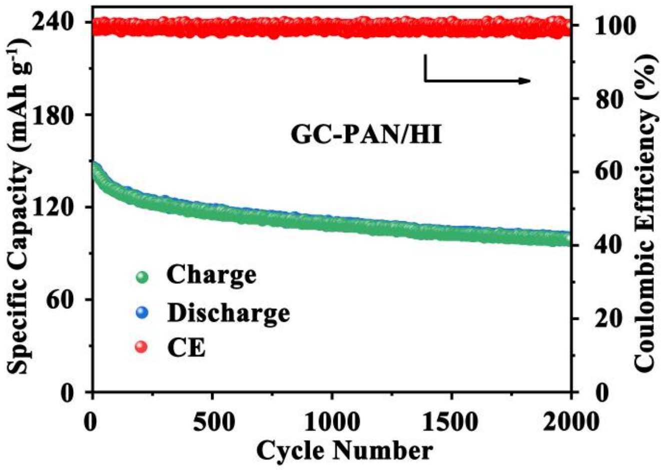

Figure S14. Long-term cycling performance of GC-PAN/HI cathode at 10 C.

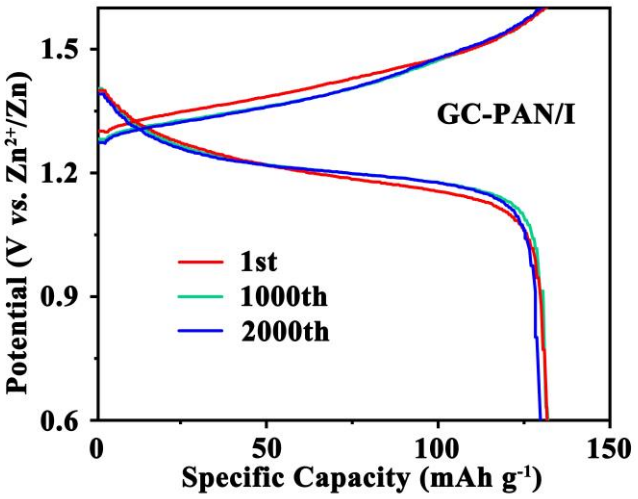

Figure S15. The corresponding galvanostatic discharge/charge profiles of GC-PAN/I at 10 C.

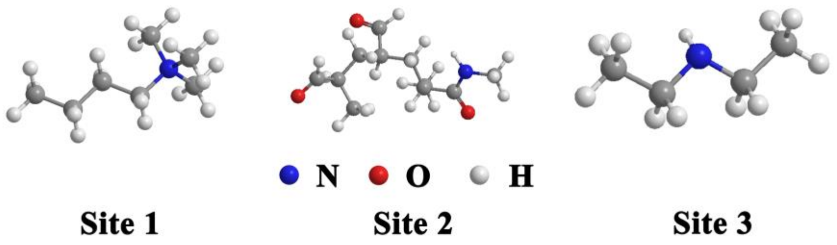

Figure S16. GC-PAN/I was divided into three slices, namely Site 1-quaternary ammonium, Site 2-amide, and Site 3-secondary amine.

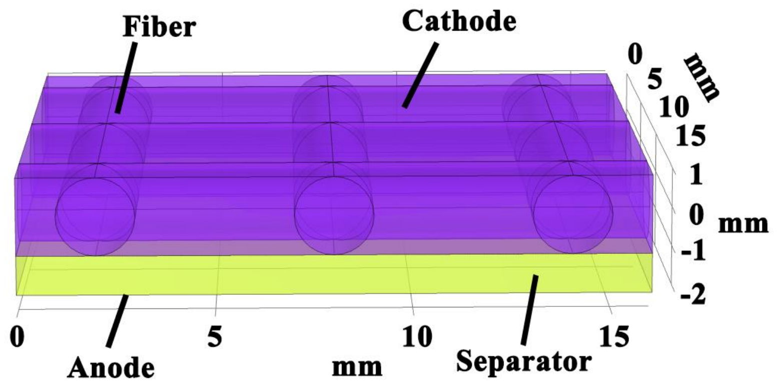

Figure S17. The 3D geometric construction of Zn-I_2 battery with GC-PAN/I cathode by COMSOL Multiphysics simulation.

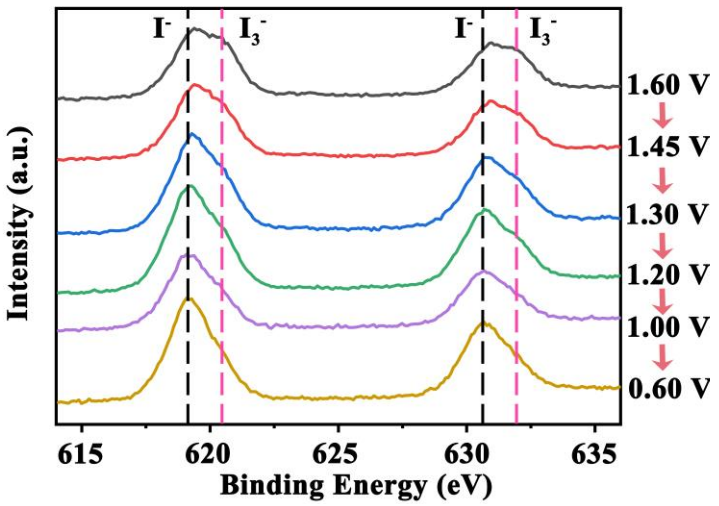

Figure S18. Ex-XPS analysis of GC-PAN/I electrode during the discharge process from 1.6 to 0.6 V. Remarkably, it just refers to a conversion reaction between I_3^- and I^-.

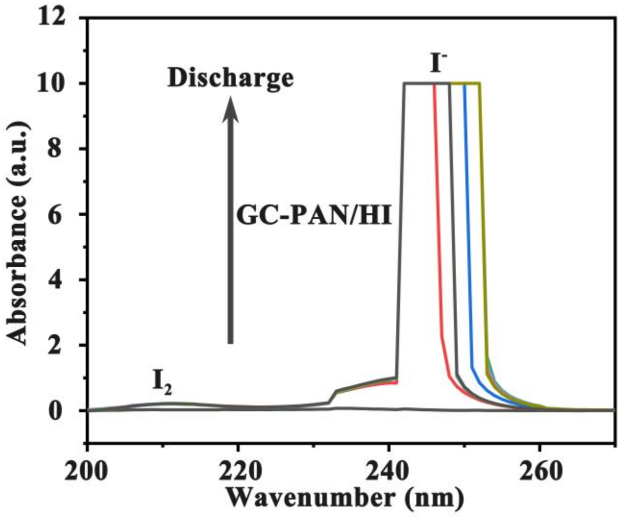

Figure S19. In-situ UV-vis analysis of Zn-I_2 batteries with GC-PAN/HI electrode during the discharge process. Apparently, an I_2 characteristic peak presents at about 210 nm, indicating a solid-liquid reaction mechanism in GC-PAN/HI electrode. Owing to the weak interaction between GC-PAN/HI and polyiodide, the discharge product of I^- quickly immerses into the electrolyte, causing its concentration to exceed the detection limits.

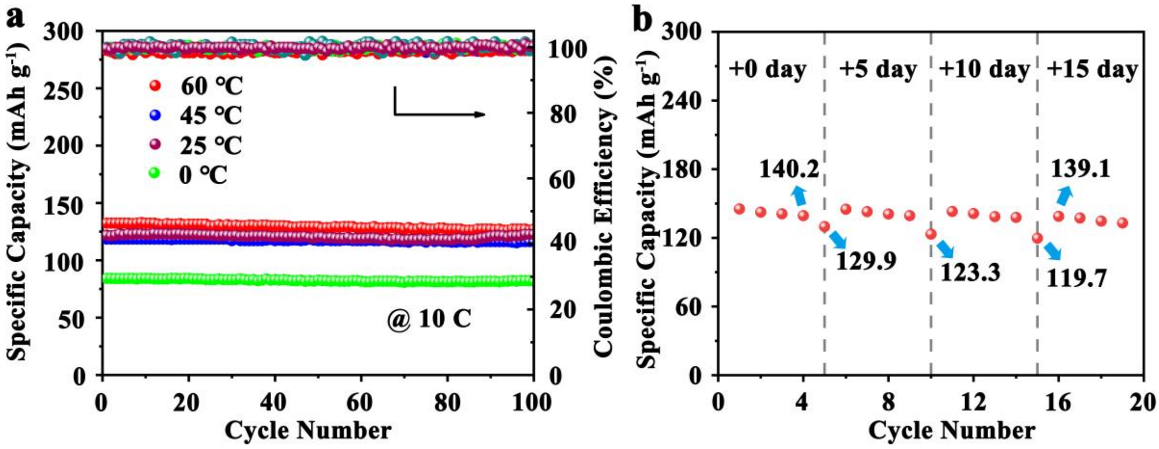

Figure S20. a) Stability performance of the GC-PAN/I cathode at different temperatures from 0 to 60 °C at 10 C. b) Self-discharge profile and the stability performance of GC-PAN/I-based Zn-I_2 cell with different resting times of 5, 10, and 15 d.

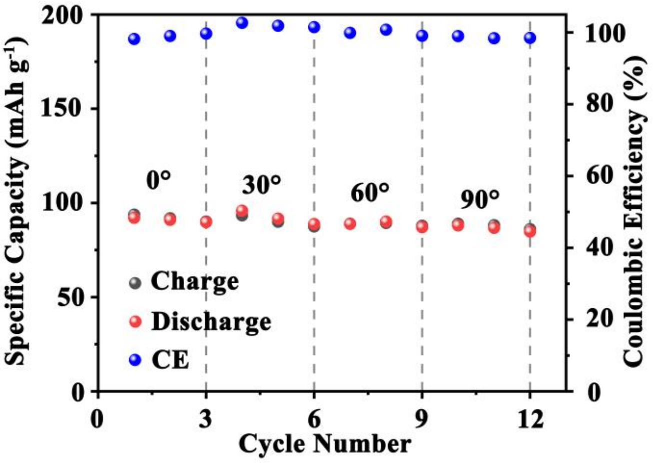

Figure S21. Cycling stability of quasi-solid-state Zn-I_2 battery with GC-PAN/I cathode at different bending angles of 0°, 30°, 60°, and 90°.

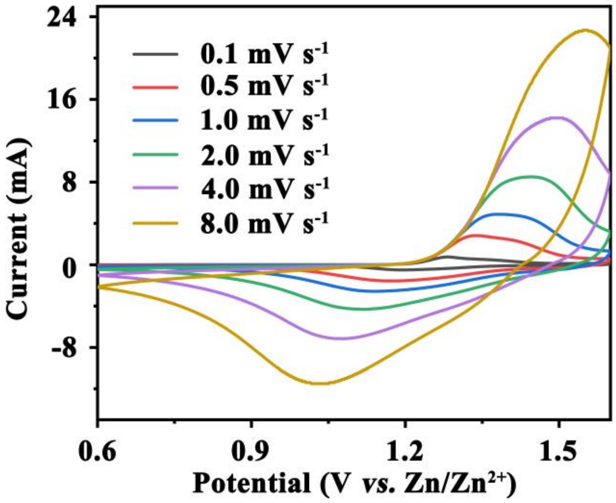

Figure S22. CV curves of the QR-D296/I electrode at different scan rates from 0.1 to 8.0 mV s^{-1}.

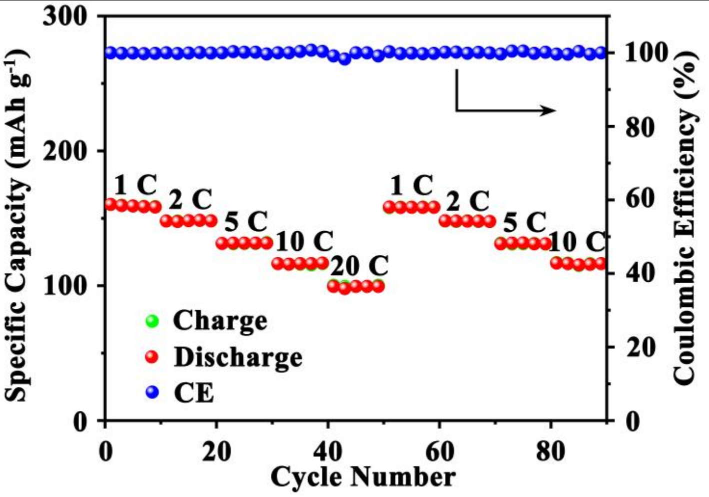

Figure S23. Rate performance of the QR-D296/I electrode at different C-rates from 1 to 20 C.

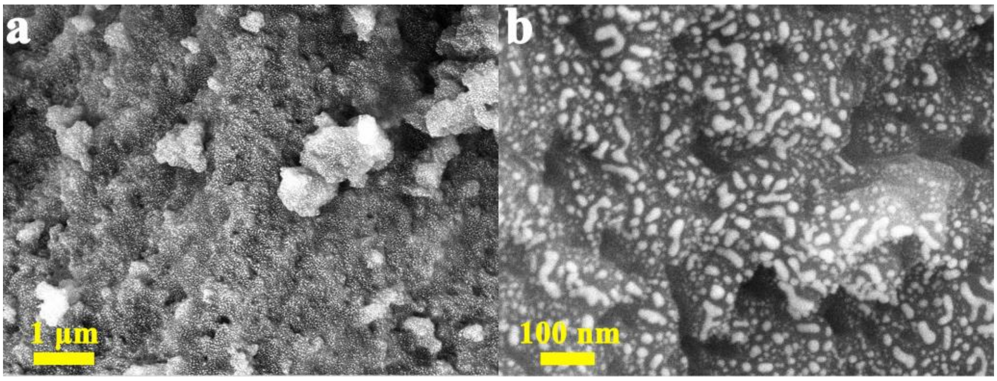

Figure S24. The SEM images of QR-D296/I at different scales of a) 1 μm and b) 100 nm.

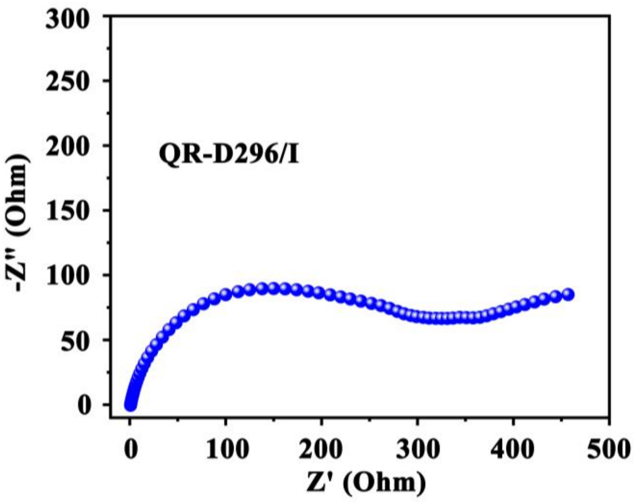

Figure S25. EIS measurement for the Zn-I_2 cell with the QR-D296/I cathode.

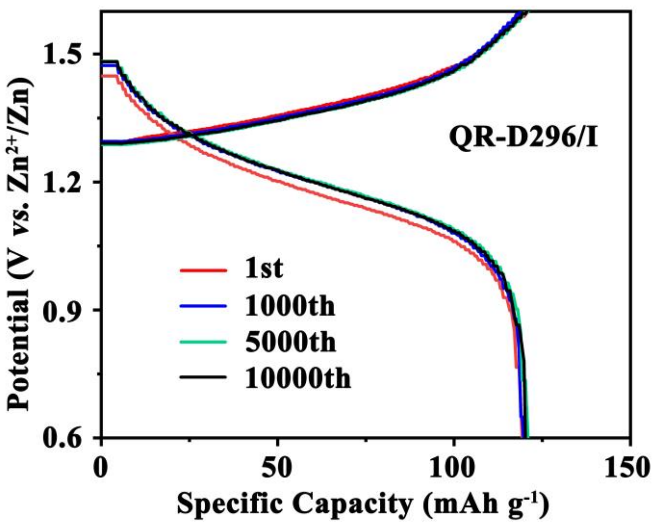

Figure S26. The corresponding galvanostatic discharge/charge profiles of QR-D296/I at 10 C.

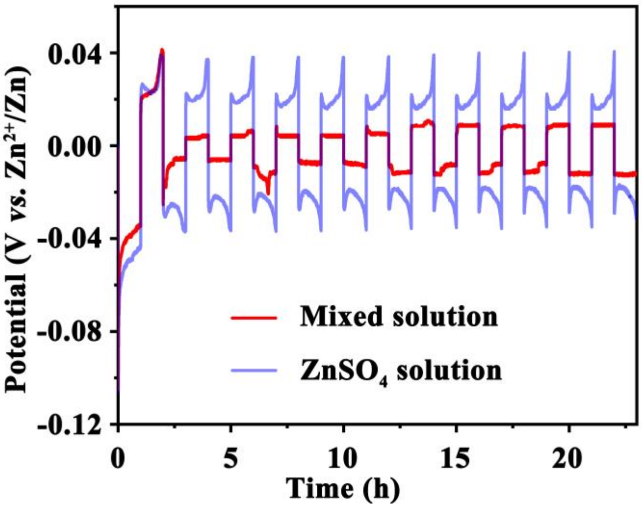

Figure S27. The symmetrical Zn cells with the mixed electrolytes (polyiodide (10 vol%, composed of 1 M KI and 0.01 M I_2 in deionized water) and 2 M $ZnSO_4$ (90 vol%)), and 2 M ZnSO4 at a current density of 5 mA cm^{-2} (5 mAh cm^{-2}).

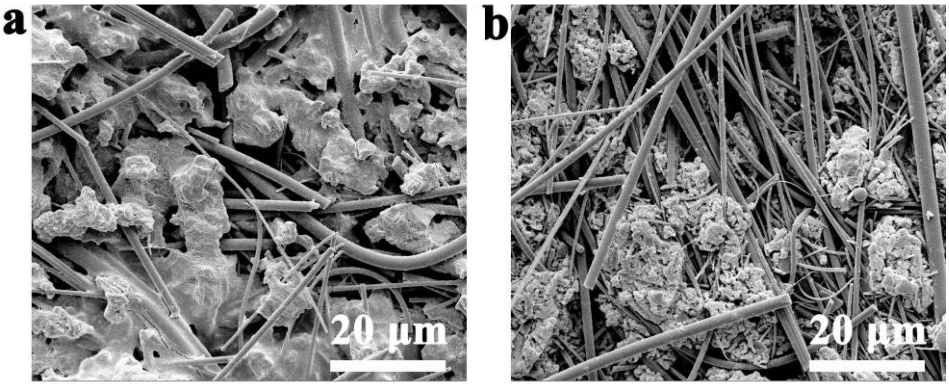

Figure S28. The SEM images of Zn anode after cycling with a) mixed electrolyte, and b) 2 M $ZnSO_4$ electrolyte.

Part II: Supporting Tables

Table S1. The calculated binding energies of Site 2 and Site 3 to different iodine species in GC-PAN/I cathode.

	I^-	I_3^-	I_2
Site 2	-0.63 eV	-0.69 eV	-0.34 eV
Site 3	-0.29 eV	-0.22 eV	-0.84 eV

Table S2. Relative literature values of zinc-iodine cells are compared with this work.

**Materials**	**Cycle number**	**Current density ($mA\ g^{-1}$)**	**Capacity retention (%)**	**Ref.**
Self-assembled solid-State gel catholyte	500	210	94	9 (9)
Three-dimensional graphene foams co-doped with sulfur and nitrogen	500	200	79	10 (15)
Iodine/carbon cloth cathode	200	227	91	11 (21)
Microporous carbon/iodine composite	3000	422	90	12 (22)
Prussian blue analogue hosts	2000	4000	80.2	13 (23)
The polyiodide doped conductive polymer	700	1500	79	14 (32)
I-terminated halogenated $Ti_3C_2I_2$ MXene	2800	3000	80	15 (33)
Nanoporous carbon cloth substrate/iodine composite	1500	1055	90	16 (34)
Modified acrylic fiber/iodine composite (GC-PAN/I)	2000	3200	97	This work

Note: in the column of Ref., the first number represents the serial number of Ref. in the Supporting Information, and the number in the bracket refers to the serial number of Ref. in the main text.

References

[1] X. Deng, J. Li, Z. Shan, J. Sha, L. Ma, N. Zhao, *J. Mater. Chem. A* **2020**, *8*, 11617.
[2] C. Adamo, V. Barone, *J. Chem. Phys*. **1999**, *110*, 6158.
[3] F. Weigend, R. Ahlrichs, *Phys. Chem. Chem. Phys*. **2005**, *7*, 3297.
[4] F. Weigend, *Phys. Chem. Chem. Phys.* **2006**, *8*, 1057.
[5] S. Grimme, S. Ehrlich, L. Goerigk, *J. Comp. Chem.* **2011**, *32*, 1456.
[6] Gaussian 16, Revision A.03, M. J. Frisch, G. W. Trucks, H. B. Schlegel, G. E. Scuseria, M. A. Robb, J. R. Cheeseman, G. Scalmani, V. Barone, G. A. Petersson, H. Nakatsuji, X. Li, M. Caricato, A. V. Marenich,J. Bloino, B. G. Janesko, R. Gomperts, B. Mennucci, H. P. Hratchian, J. V. Ortiz, A. F. Izmaylov, J. L. Sonnenberg, D. Williams-Young, F. Ding, F. Lipparini, F. Egidi, J. Goings, B. Peng, A. Petrone, T. Henderson, D. Ranasinghe, V. G. Zakrzewski, J. Gao, N. Rega, G. Zheng, W. Liang, M. Hada, M. Ehara, K. Toyota, R. Fukuda, J. Hasegawa, M. Ishida, T. Nakajima, Y. Honda, O. Kitao, H. Nakai, T. Vreven, K. Throssell, J. A. Montgomery, Jr., J. E. Peralta, F. Ogliaro, M. J. Bearpark, J. J. Heyd, E. N. Brothers, K. N. Kudin, V. N. Staroverov, T. A. Keith, R. Kobayashi, J. Normand, K. Raghavachari, A. P. Rendell, J. C. Burant, S. S. Iyengar, J. Tomasi, M. Cossi, J. M. Millam, M. Klene, C. Adamo, R. Cammi, J. W. Ochterski, R. L. Martin, K. Morokuma, O. Farkas, J. B. Foresman, and D. J. Fox, Gaussian, Inc., Wallingford CT, **2016**.
[7] K. Kumaresan, Y. Mikhaylik, R. E. White, *J. Electrochem. Soc.* **2008**, *155*, A576.
[8] M. Ghaznavi, P. Chen, *J. Power Sources* **2014**, *257*, 394.
[9] K. K. Sonigara, J. Zhao, H. K. Machhi, G. Cui, S. S. Soni, *Adv. Energy Mater.* **2020**, *10*, 2001997.
[10] K. Lu, H. Zhang, B. Song, W. Pan, H. Ma, J. Zhang, *Electrochim. Acta* **2019**, *296*, 755.
[11] Y. Li, L. Liu, H. Li, F. Cheng, J. Chen, *Chem. Commun.* **2018**, *54*, 6792.
[12] H. Pan, B. Li, D. Mei, Z. Nie, Y. Shao, G. Li, X. S. Li, K. S. Han, K. T. Mueller, V. Sprenkle, J. Liu, *ACS Energy Lett*. **2017**, *2*, 2674.
[13] L. Ma, Y. Ying, S. Chen, Z. Huang, X. Li, H. Huang, C. Zhi, *Angew. Chem. Int. Ed.* **2021**, *60*, 3791.
[14] X. Zeng, X. Meng, W. Jiang, J. Liu, M. Ling, L.Yan, C. Liang, *ACS Sustainable Chem. Eng.* **2020**, *8*, 14280.
[15] X. Li, M. Li, Z. Huang, G. Liang, Z. Chen, Q. Yang, Q. Huang, C. Zhi, *Energy Environ. Sci.* **2021**, *14*, 407.
[16] C. Bai, F. Cai, L. Wang, S. Guo, X. Liu, Z. Yuan, *Nano Res*. **2018**, *11*, 3548
